# Tomato molecular biology – special collection of papers for molecular horticulture

**DOI:** 10.1186/s43897-022-00042-z

**Published:** 2022-09-20

**Authors:** Graham B. Seymour, Jocelyn K. C. Rose

**Affiliations:** 1 School of Biosciences, Division of Plant and Crop Science, University of Nottingham, Loughborough, Leics LE12 5RD UK; 2 School of Integrative Plant Science, Cornell University, 331 Emerson Hall, Ithaca, NY 14853 USA

Tomato (*Solanum lycopersicum*) is the second most important vegetable crop globally, after potato, with about 100 million tons fresh fruit being grown on 3.7 million hectares (FAO 2021), and is of great importance in the human diet due to the large amount of fruit consumed. The fruit are eaten both fresh and, equally importantly, as a processed product in puree, soups and canned products. They provide an important dietary source of vitamins and minerals, such as K, Fe and Ca, and are known for the large number of health promoting secondary metabolites, including the carotenoid and flavonoid pigments that give the fruit a spectrum of yellow, red and orange colours.

Tomato commercial production and breeding is supported by a long history of research that has led to the identification of regions of the tomato genome that control a host of important traits including disease resistance, yield and fruit quality. The first tomato genome was sequenced a decade ago and the sequence is now in its fifth iteration with 100 s of genome sequences from a wide spectrum of wild species crop relatives and cultivated varieties. The diploid nature of tomato, genomic resources and a wide range of single gene mutants make it an excellent model plant to study dicot crop species and especially those with fleshy fruits.

In this special collection, we bring together a range of papers to explore the latest developments and scientific insights in tomato molecular biology. This brief editorial summarises the key features of the papers that collectively set the scientific discovery in the context of plant development and highlight relevance to horticulture.

Plant architecture is a critical determinant of crop productivity and influences, among other factors, the number of reproductive shoots. The hormonal control of plant architecture has been the subject of studies for well over 100 years, but recent advances in genomics have accelerated our understanding of its molecular basis. Tomato is an especially useful model to investigate the association between plant architectural features and their impact on crop performance. Side branching in tomato is undesirable and results in unwanted labour and management costs. Three main phytohormone classes are known to influence side branching: auxins, cytokinins (CKs) and strigolactones. Auxin inhibits the outgrowth of axillary buds and maintains apical dominance. CKs act antagonistically to auxin, suppressing apical dominance and allowing release of axillary buds from dormancy. Auxin modulates CK concentration by repressing its levels, while strigolactones affect bud inhibition by modulating auxin transport. In addition, there seems to be some interplay between CK and strigolactone levels, and the overall picture highly complex. In this special issue, Pino et al. (2022) report that tomato plants overexpressing the cytokinin-deactivating gene *CYTOKININ OXIDASE 2* (*CKX2*) showed excessive growth of axillary shoots, which is opposite to the phenotype expected of plants with reduced CK content. The authors suggest that CKs cause their paradoxical effects on branching by disturbing auxin status, and by altering the expression of genes associated with branching and CK homeostasis. The study highlights the intricacy of the molecular control of side branching, and the importance of this research in understanding the control of plant architecture for crop improvement.

The architecture of plant roots is as important as that of above ground organs and this is an area of particular interest to growers of grafted vegetable crops. Many Solanaceous crops, including tomato, pepper and eggplant, are grown as elite scion genotypes grafted onto to superior performing rootstocks, and this is especially widespread for commercial tomato production in Europe and the USA. The root stocks can be chosen to enhance scion resistance to diseases and abiotic stress, involving conditions such as low nutrient availability and high salinity. The genetic basis of root traits in tomato and other crops is relatively poorly understood and this knowledge is an important prerequisite for a rational approach to breeding for improved root traits. In this issue, Kevei et al. (2022) report their work on a tomato mutant, *bushy root-2* (*brt-2*), which has a twisting tap root and a high density of lateral roots, giving a bushy appearance. These lateral roots are also abnormal in that they twist and curl, and plant growth is slower than that of wild type. The *brt-2* candidate gene was identified by genetic mapping as a class B heat shock factor protein encoded by SolycHsfB4a. Whole genome resequencing and SNP (single nucleotide polymorphism) and KASP (Kompetitive allele specific PCR) markers were used to fine map the *brt-2* gene and a SNP was identified as a strong candidate for the causal *brt- 2* mutation. The authors discuss reasons why such a mutation could influence the function of the protein encoded by the gene at the *brt-2* locus and how this might result in the bushy root phenotype. The study provides an important extension to our knowledge of root architecture in tomato. Moreover, since a related *Arabidopsis thaliana* gene, *ATHSFB4*, is induced by root knot nematode (RKN) infection, and its loss-of-function mutants are resistant to RKNs, *BRT-2* could be a target gene for RKN resistance, an important trait in tomato rootstock breeding.

Studies have shown that the action of auxin, CKs, and gibberellins (GAs) can reduce plant resistance to water deficiency, and there is evidence that inhibition of GA activity can enhance plant performance under stress conditions. In this collection, Shohat et al. (2021) review the importance of GAs in tomato, including in regulating responses to abiotic stresses, such as drought. They authors consider how drought affects GA biosynthesis and signalling in tomato and discuss possible ways in which knowledge of GA pathways could be used to generate drought tolerant plants. These include interrupting GA binding to its receptor, GID1, altering the effectiveness of the downstream signalling pathway and, perhaps most promisingly, through deactivation of GA itself.

Irregular watering and other factors can result in devastating losses in commercial tomato operations due to a physiological disorder known as blossom-end rot (BER). Typical symptoms of BER appear as small, light-coloured, water-soaked spots on the blossom end of the fruit, which is associated with cell plasmolysis and leaky membranes. After BER induction, BER-affected areas often expand in the form of brown necrotic regions covering a significant proportion of the fruit and, in some extreme cases, can affect the entire fruit. The condition affects tomato, but also many other fruits, including pepper (*Capsicum annuum* L.), watermelon (*Citrullus lanatus* (Thunb.) and eggplant (*Solanum melongena* L.). In this special collection, Topcu et al. (2022) review the latest information of the biological basis of BER as well as the genetic and molecular underpinnings of this important physiological disorder. The paper presents information on the role of altered Ca^2+^ homeostasis among different cellular compartments, and especially the role of Ca^2+^ and interactions with pectin in the fruit primary cell walls. Other important factors include reactive oxygen species (ROS), which appear to be a critical component of BER development and are linked to changes in Ca^2+^ deficiency. Tomato varieties show variation in their susceptibility to BER, which suggests a possible underlying genetic basis for the condition. Genetic mapping and differential expression analysis has uncovered possible candidate BER-associated genes, but further work is needed to develop genetic approaches to prevent this physiological disorder.

Tomato fruit are the most widely used model to investigate the developmental regulation of ripening in fleshy fruited species, and there is a deep set of resources to help dissect the biochemical, molecular, and genetic events linked to tomato fruit ripening control. In this special collection, Zhu et al. (2021) review the metabolic changes that effect fruit quality during tomato ripening. The review focuses on the transcriptional and post-translational control of the networks that affect the accumulation of important biochemical components in fruit tissues, from pigments and sugars through to other metabolites with health promoting properties. The review links the biochemistry of the ripening process with the key genes underlying these processes and the associated quality traits.

One of the key processes determining fruit quality is the rate of softening and tomato again represents one of the model organisms where this process has been researched in some considerable depth. The review in this collection by Wang and Seymour (2022) summarises the most recent data available on the control of softening in tomato. The authors first describe the hormonal cues, epigenetic priming and transcriptional control linked with the softening process. These areas of research are still fragmented and there is a limited understanding of the interrelationship between such high-level events. However, the actual biochemical changes involved in texture changes are a little clearer, although the specific timing and role of particular biochemical events still generally remain obscure. In essence, it seems that modification of pectic polysaccharides plays a major role in softening through the action of enzymes such as pectate lyase (PL), polygalacturonase (PG) and several other activities. Gene editing of tomato to silence these genes leads to an inhibition of softening in the case of PL, although it is not the only factor involved in textural changes and studies of the transcriptional control of softening are providing new insights. Understanding the molecular basis of softening is providing targets for molecular breeding that will likely improve shelf life, and possibly fruit that are more resilient to fungal and bacterial spoilage.

A primary barrier to pathogen invasion and spoilage is the fruit cuticle. In this issue Bres et al. (2022) describe how they screened a mutant collection of the miniature tomato cultivar Micro-Tom for fruit cracking mutants and found a mutant with a glossy fruit phenotype. The authors then used a mapping-by-sequencing strategy to identify the causal mutation as an amino acid change in the SlSHN2 transcription factor, which is specifically expressed in outer epidermis of growing fruit. The mutation has a marked effect on cuticle composition. In addition to the direct effects on cuticle formation and composition, the mutation resulted in a wide range of other gene expression changes that link the *SlSHN2* gene to coordination of cuticle deposition, epidermal patterning and defence against biotic and abiotic stresses.

New insights into the molecular control of plant growth and fruit ripening will likely come with a better understanding of the role of genome structural variation and epigenetics in controlling gene expression. The paper in this special collection by Jobson and Roberts (2022) reviews the current understanding of genomic structural variation (SV) in tomato and its role in plant immunity. Structural variation for the purpose of this review is defined as changes that range from greater than 30 base pairs to several megabases. These can include inversions, duplications and deletions. The authors discuss the various molecular mechanisms leading to common structural changes. Drawing on information from several species, including tomato, they then discuss identification of SVs in plant genomes. The main part of the review focuses on the potential roles of SV in responses to biotic and abiotic stress. The authors conclude with a section on engineering immunity in tomato using SVs.

Other articles published in Molecular Horticulture, outside this current collection that may also be of interest to readers include those on tomato fruit size control by a zinc finger protein regulating pericarp cell size (Zhao et al. 2021), tomato SlRUP as a negative regulator of UV-B photomorphogenesis (Zhang et al. 2021) and genome-wide binding analysis of the tomato transcription factor SlDof1 and its regulatory impacts on fruit ripening (Wang et al. 2021).

The papers in this special collection highlight a broad range of exciting discoveries, technology platforms and resources that illustrate the tremendous and growing significance of tomato as an experimental model, and socially important crop. The future for tomato research remains bright!
